# Supplementary material for: Mouse microglia express unique miRNA-mRNA networks to facilitate age-specific functions in the developing central nervous system
Source: Commun Biol. 2023 May 22;6:555. doi: 10.1038/s42003-023-04926-8 (PMC10203306; doi:10.1038/s42003-023-04926-8)
Supplement: Supplementary file 2 — Supplementary figures [file 42003_2023_4926_MOESM2_ESM.pdf]

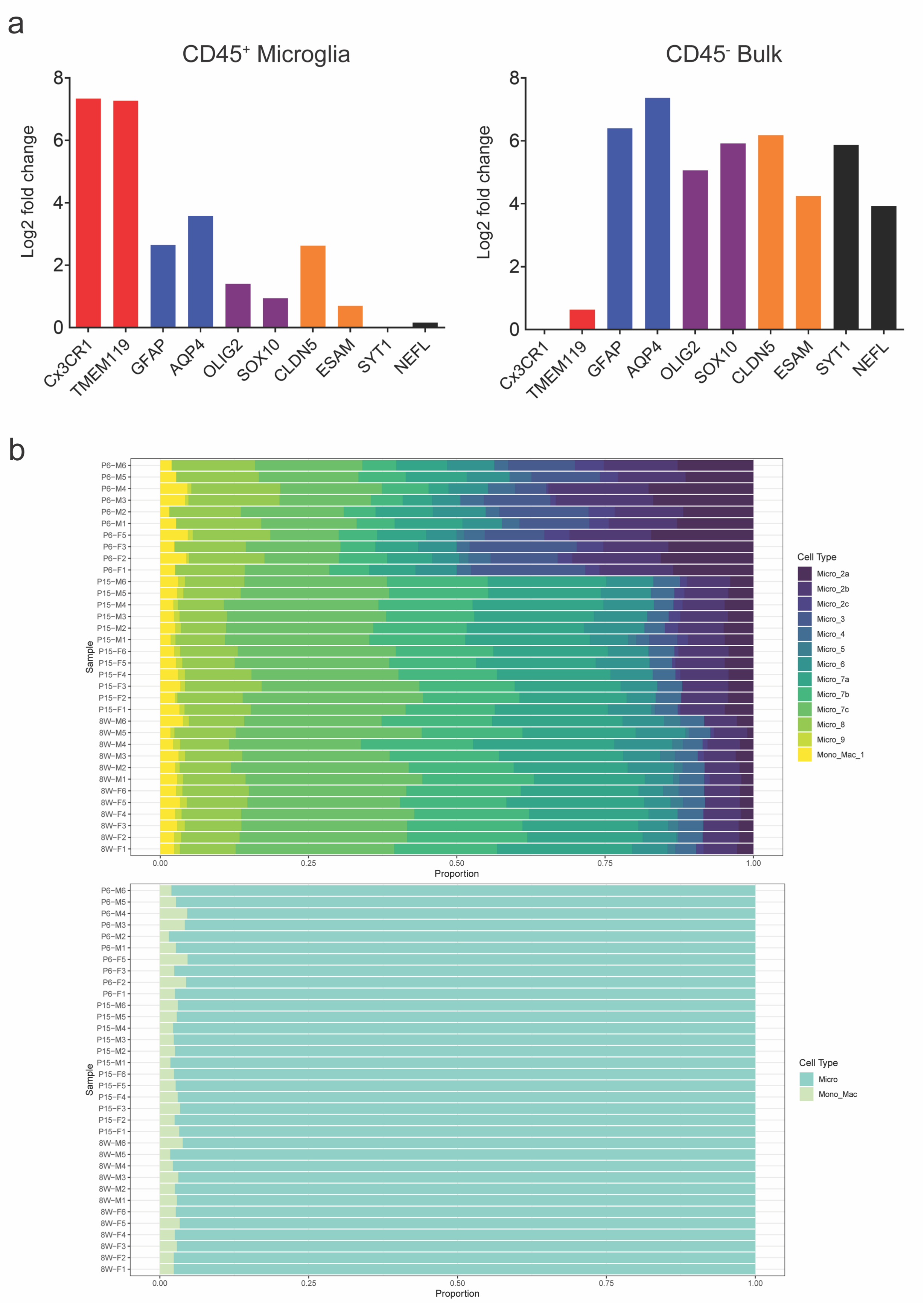
a
CD45+ Microglia
CD45- Bulk
Log2 fold change
Cx3CR1
TMEM119
GFAP
AQP4
OLIG2
SOX10
CLDN5
ESAM
SYT1
NEFL
b
Sample
Proportion
Cell Type
Micro_2a
Micro_2b
Micro_2c
Micro_3
Micro_4
Micro_5
Micro_6
Micro_7a
Micro_7b
Micro_7c
Micro_8
Micro_9
Mono_Mac_1
Micro
Mono_Mac

Supplementary Figure 1: Validation of CD45^{+ve} immunopanning technique via qPCR and cell proportion estimation with CIBERSORTx

a. qPCR of 10 target genes from CD45^{+ve} and CD45^{-} immunopanning samples from a P6 mouse (n=1, mean of three technical replicates). Gene targets represent major cell types of the CNS; Microglia (CX3CR1,TMEM119), Astrocytes (GFAP, AQP4), Oligodendrocytes/OPCs (OLIG2, SOX10), Endothelial Cells (CLDN5, ESAM), Neurons (SYT1, NEFL). CD45^{+} populations exhibit strong enrichment of microglial gene targets and low contamination of other cell types. Conversely, the CD45^{-ve} sample exhibits expression of all other cell types and very low contamination of microglial cells. b. RNAseq gene expression from bulk immunopanned samples were compared against a single cell microglial dataset derived from CX3CR1 FACS sorted cells across the mouse lifespan[31]. Top panel shows proportions of all 12 identified microglial subsets (Micro) and single non microglial monocyte/macrophage subset (Mono_Mac) from Hammond et al. 2019. Bottom panel shows all microglial subsets combined compared to Mono_Mac. CIBERSORTx analysis observed an average of 2.8% non microglial (monocyte/macrophage) contamination per sample.

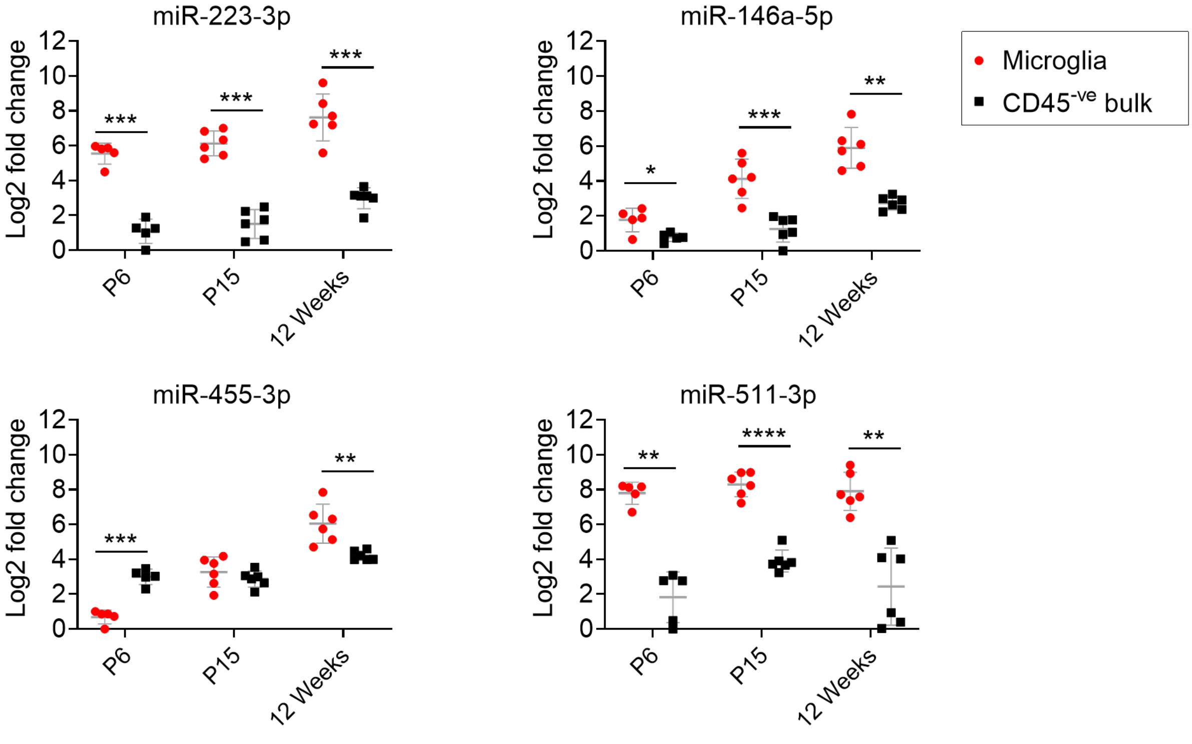

Supplementary Figure 2: qPCR validation of age specific miRNA expression. Expression of 4 miRNA candidates (223-3p, 146a-5p, 455-3p, 511-3p) were assessed in P6, P15 and adult mice (12 weeks) (n=5-6 per group). Data were analysed by paired t test comparing microglia and CD45^{-ve} bulk expression within each age group. * $p < 0.05$, ** $p < 0.01$, *** $p < 0.001$, **** $p < 0.0001$. qPCR data is presented as mean ± SD.

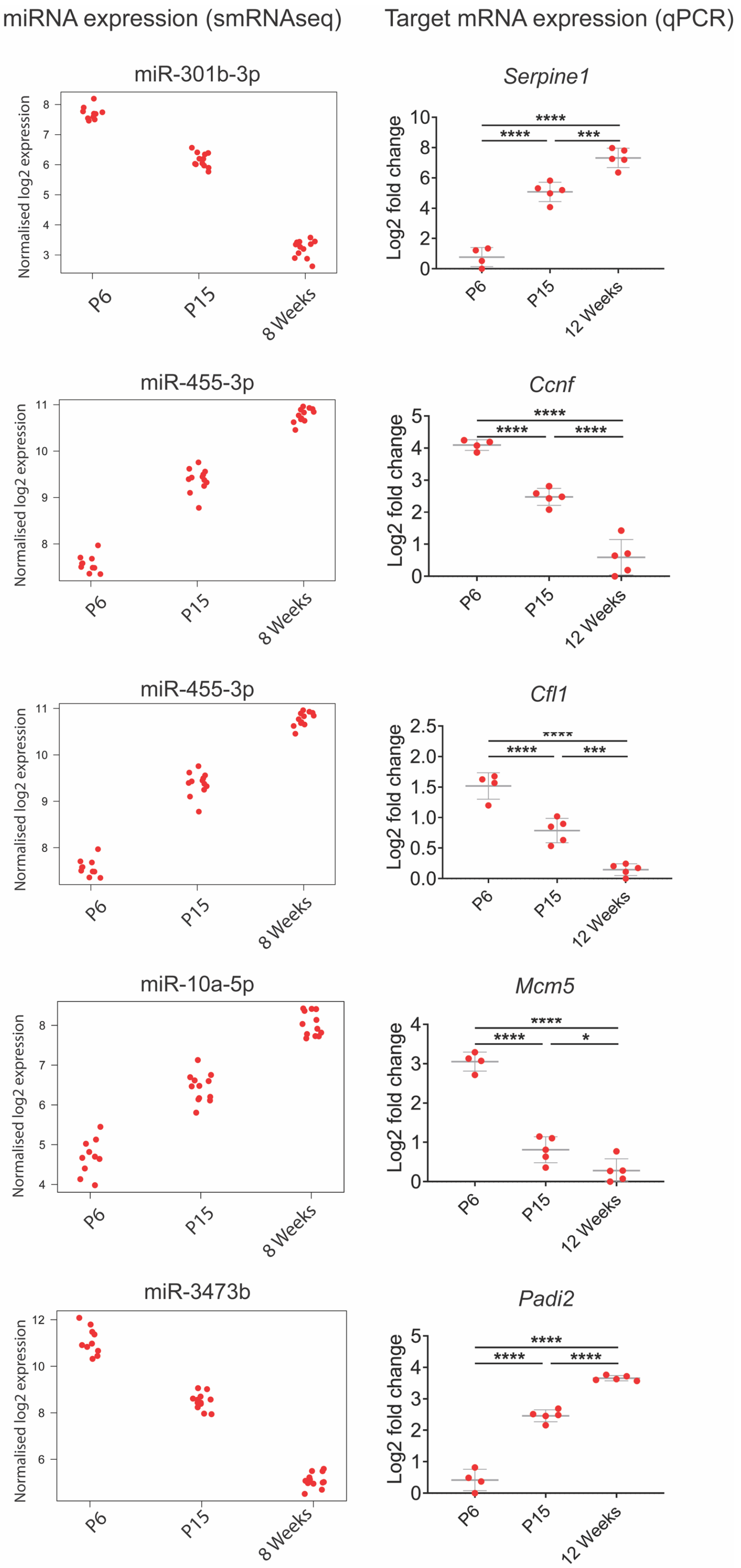

miRNA expression (smRNAseq)
Target mRNA expression (qPCR)
miR-301b-3p
Serpine1
miR-455-3p
Ccnf
miR-455-3p
Cfl1
miR-10a-5p
Mcm5
miR-3473b
Padi2
Normalised log2 expression
Log2 fold change
P6
P15
8 Weeks
12 Weeks
****
***
*

Supplementary Figure 3: qPCR validation of mRNA target genes.

smRNAseq expression data for 6 candidate miRNAs and qPCR expression data for their predicted mRNA targets (based on negative correlation and experimental evidence of interaction, refer to Methods). All tested targets exhibited a negative correlation of expression with their putative miRNA binding partners. PCR data were generated in P6, P15 and adult mice (12 weeks) (n=4-5 per group). Data were analysed using one-way Anova followed by uncorrected Fisher's LSD. * $p < 0.05$, ** $p < 0.01$, *** $p< 0.001$, **** $p < 0.0001$. qPCR data is presented as mean ± SD.

Supplementary Table 1: Top expressed miRNAs and mRNAs in Microglia

Top expressed miRNAs and mRNAs in Microglia

Top 15 expressed miRNAs/mRNAs by average expression across all ages

miRNA					mRNA				
miRNA	P6	P15	8W	Average	mRNA	P6	P15	8W	Average
let-7c-5p	16.47	16.09	16.13	16.23	Actb	13.18	13.02	12.84	13.01
miR-9-5p	16.84	15.83	15.51	16.06	Csf1r	11.97	13.25	13.26	12.82
miR-181a-5p	15.30	15.91	16.42	15.88	Cst3	11.19	13.22	13.82	12.74
let-7a-5p	15.51	15.55	15.77	15.61	COX1	12.55	12.47	12.69	12.57
let-7f-5p	14.85	15.33	15.94	15.38	Hexb	11.66	12.87	13.15	12.56
miR-16-5p	14.64	15.29	15.42	15.12	Eef1a1	12.89	12.59	12.20	12.56
let-7i-5p	14.75	15.23	15.29	15.09	Ctsd	11.82	12.48	12.69	12.33
miR-26a-5p	14.46	15.06	15.68	15.07	Cx3cr1	11.28	12.25	12.60	12.04
let-7b-5p	15.22	14.58	14.43	14.74	Psap	11.56	12.22	12.29	12.02
let-7g-5p	14.05	14.63	15.20	14.63	Apoe	12.28	12.05	11.36	11.90
miR-29b-3p	13.36	14.63	15.78	14.59	Sparc	10.91	12.70	12.04	11.88
miR-451a	13.37	15.33	14.42	14.37	Lgmn	11.57	12.06	11.89	11.84
miR-124-3p	14.26	14.14	14.32	14.24	Ctsb	12.35	11.55	11.49	11.80
miR-191-5p	13.41	14.26	14.86	14.18	Rhob	11.00	12.21	12.17	11.79
let-7d-5p	13.73	14.11	14.51	14.12	Serinc3	11.10	11.91	12.08	11.70

All numeric values are log2 transformed

Supplementary Table 2: miRNA enrichment in microglia

miRNA enrichment in microglia

Top 10 known upregulated miRNAs vs. CD45- CNS cell types in each age group

P6			P15			8 Weeks		
miRNA	Enrichment[1]	Avg Expression[2]	miRNA	Enrichment[1]	Avg Expression[2]	miRNA	Enrichment[1]	Avg Expression[2]
miR-511-3p	9.62	7.25	miR-511-3p	7.80	7.24	miR-6983-3p	7.38	5.94
miR-6983-3p	7.99	5.31	miR-6983-3p	7.23	6.15	miR-223-3p	6.96	11.17
miR-223-3p	7.89	11.05	miR-223-3p	7.14	11.02	miR-146a-5p	6.68	15.02
miR-223-5p	7.84	3.96	miR-223-5p	6.79	3.99	miR-223-5p	6.49	3.87
miR-511-5p	7.73	4.10	miR-511-5p	6.75	4.07	miR-511-3p	6.29	5.77
**miR-1895**	7.14	5.57	miR-6983-5p	6.13	3.17	miR-142a-5p	6.16	11.12
miR-142a-3p	6.71	13.49	miR-142a-3p	6.02	14.11	miR-142a-3p	6.05	13.76
miR-142a-5p	6.22	10.10	miR-142a-5p	5.72	10.96	miR-6983-5p	5.89	3.00
**miR-3079-3p**	6.01	2.60	miR-146a-3p	5.51	4.28	miR-146a-3p	5.83	5.10
miR-6983-5p	5.99	2.14	miR-146a-5p	5.45	13.94	**miR-7219-3p**	5.51	4.50

[1] Log2 fold change comparing microglia to bulk CNS cell expression

[2] Average expression of miRNA in all microglial samples at designated age

All numeric values are log2 transformed

Bolded miRNAs are uniquely represented in the top 10 of corresponding age group

Supplementary Table 3: novel miRNA enrichment in microglia

novel miRNA enrichment in microglia

novel miRNA discovery by miRDeep2

ID	General Enrichment[2]	Age Specific Enrichment[1] P6	P15	8 Weeks	miRDeep2 score[3]	Consensus mature sequence	Genomic coordinate[4]
chr3_29427	5.05	3.98	5.95	5.22	1100.0	ucccuguccuccaggagaua	chr3:150866217..150866276:+
chr12_10577	3.98	3.81	3.72	4.41	200.0	aauuuugagcuggaaauugagg	chr12:3341345..3341401:-
chr5_33756	3.64	5.40	3.90	1.62	900.0	uggccaaggaugagaacucuaac	chr5:23852209..23852281:+ / chr5:23710989..23711061:-
chr8_43150	3.40	5.10	3.19	1.90	2600.0	gcuggaggaugaaguaagga	chr8:109839299..109839374:+
chr2_25687	3.10	2.53	1.99	4.79	150.0	aacgcggucuuuucugaguguc	chr2:120701452..120701511:+
chr4_30965	2.95	2.55	3.65	2.64	570.0	uauucauauucuucuagccuac	chr4:46495222..46495281:+
chr15_16817	2.74	3.69	1.72	2.81	100.0	aauagcuugggacaucagcucug	chr15:36595715..36595768:-
chr2_25261	2.72	2.49	3.55	2.11	4.4	uagcacaaugugaaaagagc	chr2:84741119..84741176:+
chr7_39988	2.71	2.97	2.71	2.45	100.0	ucaggcugucaguggguagag	chr7:90055622..90055672:+
chr4_33208	2.66	3.03	3.16	1.80	86.0	ucugcccuccaugcugcaga	chr4:133865904..133865963:-
chr14_15012	2.63	2.79	2.63	2.47	200.0	cauccugccuacaccuccuaag	chr14:50929380..50929452:-
chr4_31798	2.55	3.21	2.68	1.76	2700.0	auggagagacuuugacagcuc	chr4:132310003..132310068:+
chr18_22209	2.44	5.07	3.10	−0.85	190.0	ugggggugggggccgggc	chr18:26874263..26874351:-
chr8_43564	2.14	1.88	2.69	1.86	330.0	uucagggauaaauggagucacag	chr8:21456095..21456150:- / chr8:21594043..21594098:-
chr1_2994	2.13	1.99	2.12	2.26	59.0	cacccguugcucagccacucag	chr1:165689968..165690025:-
chr5_34087	2.05	2.35	2.09	1.71	61.0	uacuucagaucuagccagcag	chr5:53661140..53661195:+
chr17_19864	1.97	5.18	1.42	−0.69	5.7	ggguggggcugggccgc	chr17:50293004..50293059:+
chr11_7315	1.87	2.17	1.71	1.73	340.0	cuuucuccugcugcccugcag	chr11:103064672..103064729:+
chr14_14675	1.81	1.05	1.47	2.90	4.9	ucgagccccacguugggcgcc	chr14:16637153..16637217:-
chr7_39643	1.79	−0.09	1.63	3.83	260.0	uggagacuucccugucagagc	chr7:48961534..48961592:+
chr6_38325	1.77	4.02	1.90	−0.60	1800.0	aggcugugaugauuggcgc	chr6:71882637..71882692:-
chr14_14933	1.59	1.31	1.18	2.28	52.0	ccagucuucggcaucagugug	chr14:40907891..40907955:-
chr9_44662	1.55	2.67	1.41	0.58	530.0	cugugggaaggaacuacaagacagc	chr9:15313906..15313985:+
chr7_39362	1.51	1.50	1.55	1.47	380.0	uagcacuuguaugggguuuuugu	chr7:28351506..28351564:+

[1] Log2 enrichment of expression between CD45+ microglia and CD45- CNS cells at specified age

[2] Overall Log2 enrichment of expression between pooled microglia and CD45- CNS cell samples

[3] log-odds probability of a sequence being a genuine miRNA hairpin versus the probability it is a background hairpin

[4] Genomic coordinates of the predicted precursor miRNA sequence. Mapped to GRCm38/mm10 genome build. +/- indicates strand. chr5_33756 and chr8_43564 map identically to 2 unique genomic regions.

Supplementary Table 4: Predicted precursor structures of microglial enriched novel miRNAs predicted by miRDeep2

miRNA_ID	Predicted pri-miRNA structure – (mature miRNA sequence highlighted red)
chr1_2994	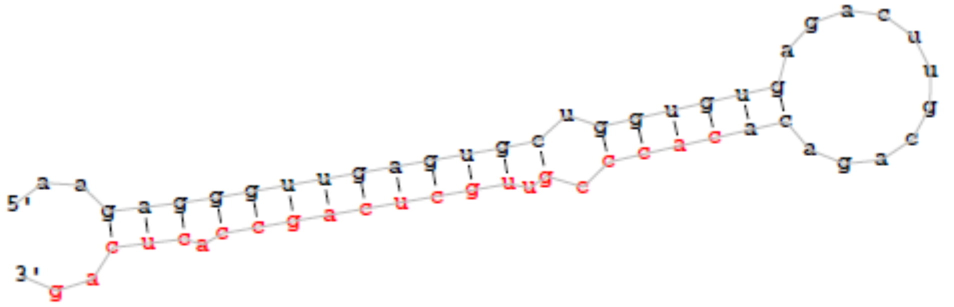
chr2_25261	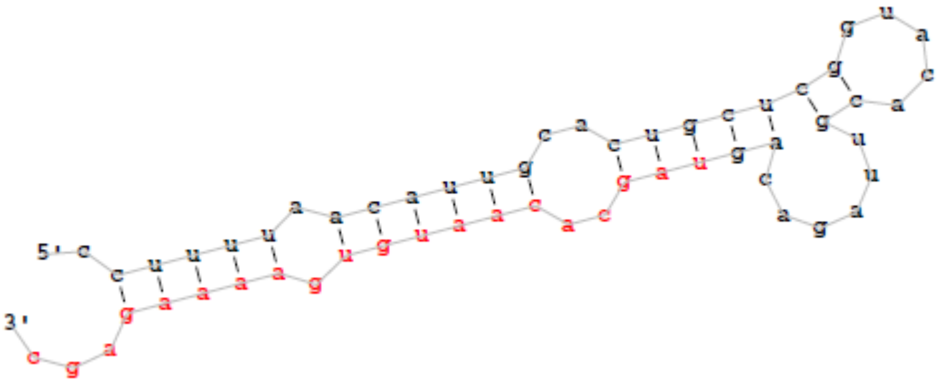
chr2_25687	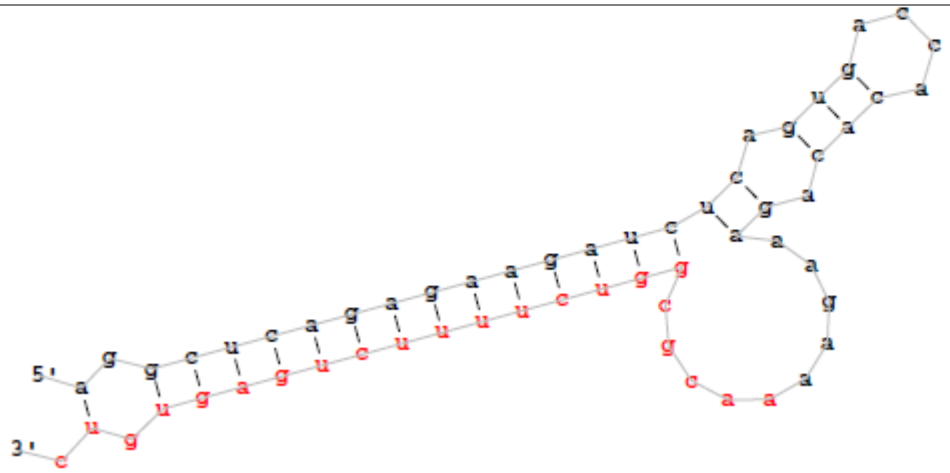
chr3_29427	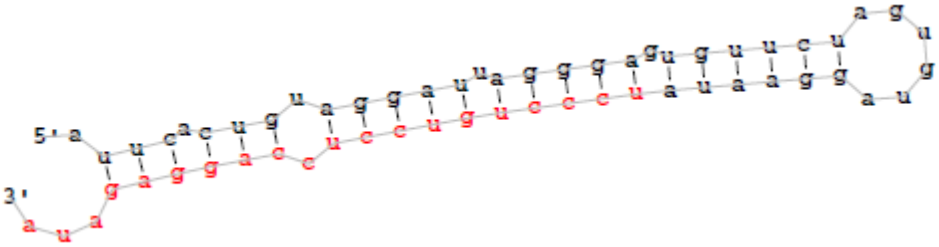

chr4_30965	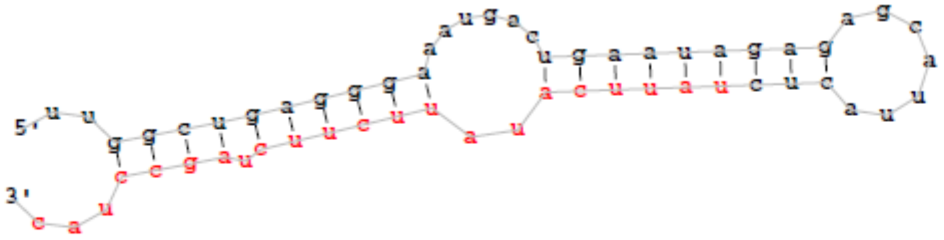
chr4_31798	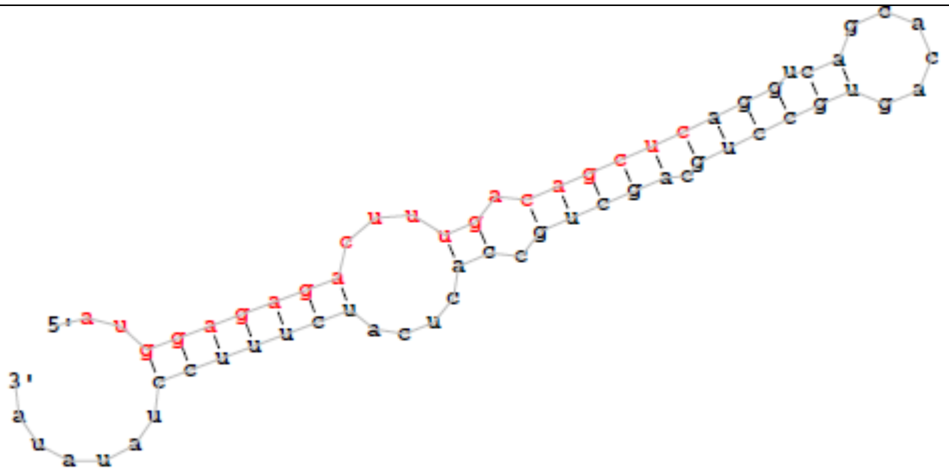
chr4_33208	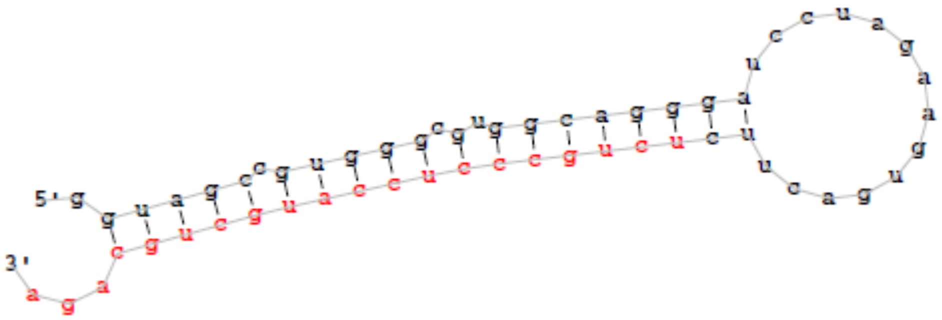
chr5_33756 *	chr5:23852209..23852281:+ 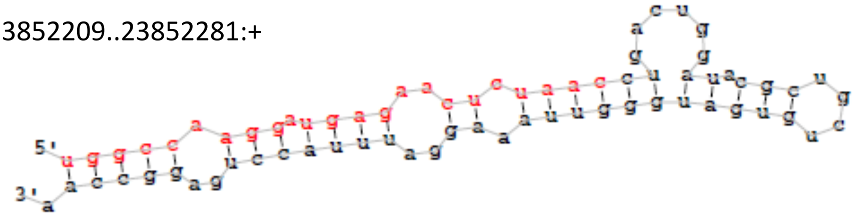 chr5:23710989..23711061:- 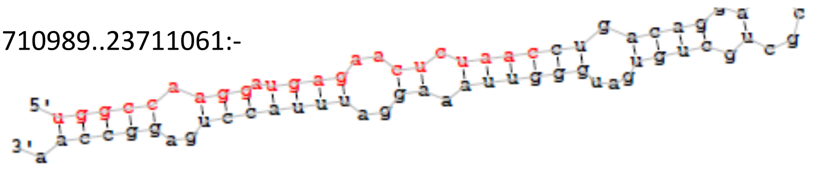

chr5_34087	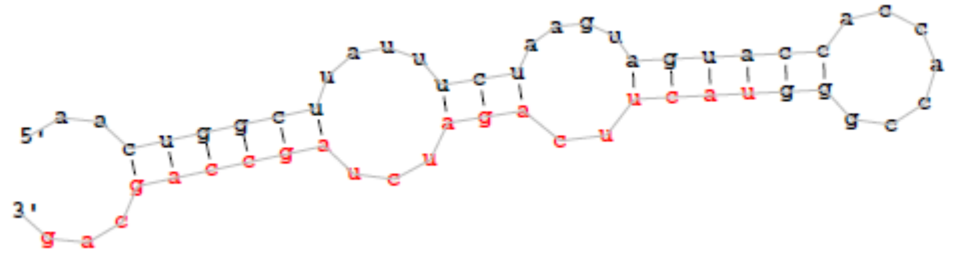
chr6_38325	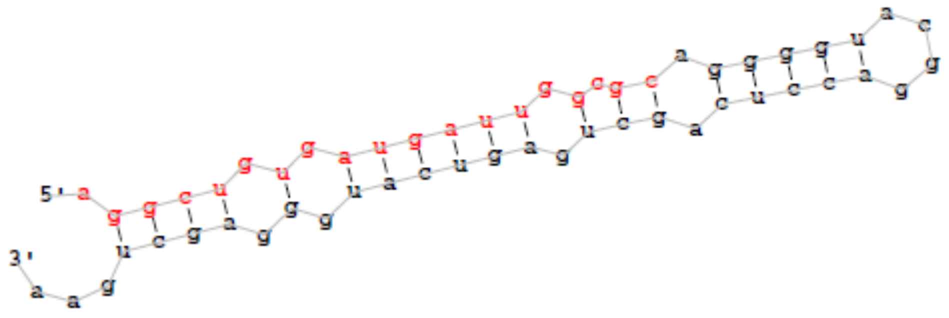
chr7_39362	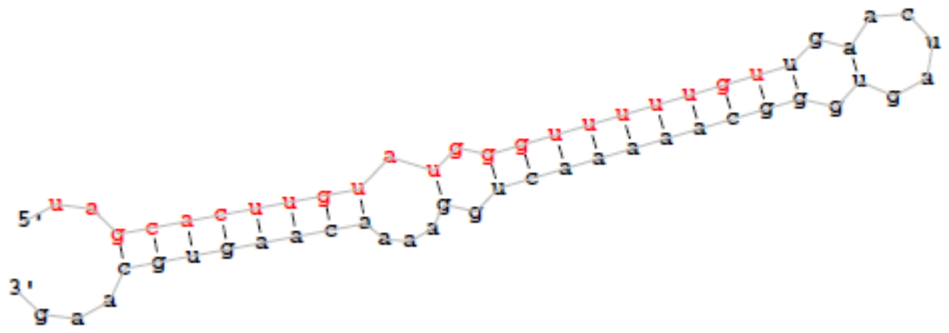
chr7_39643	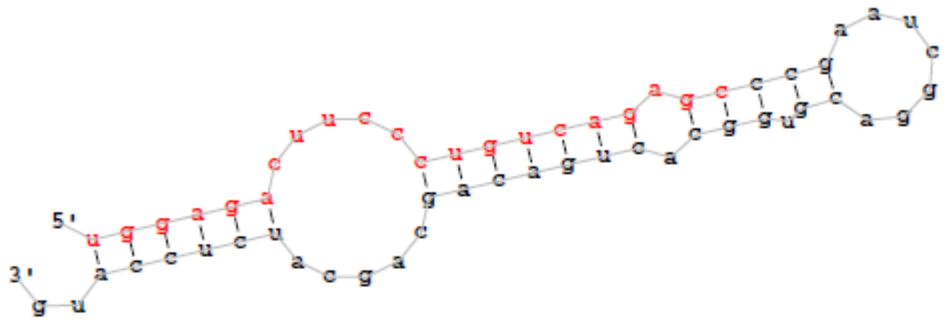

chr7_39988	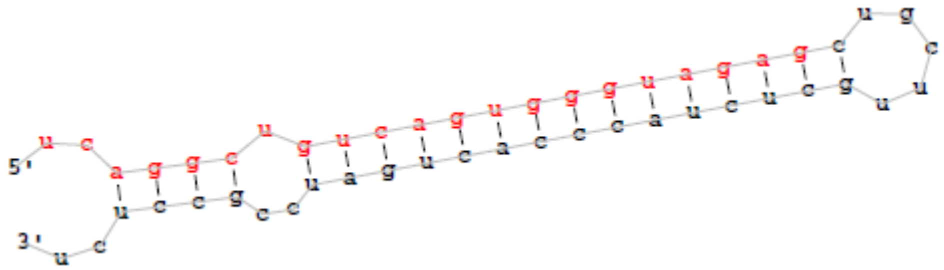
chr8_43150	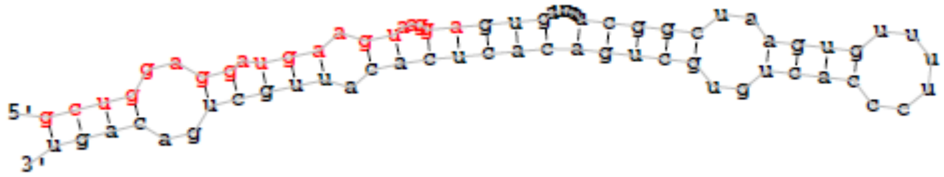
chr8_43564	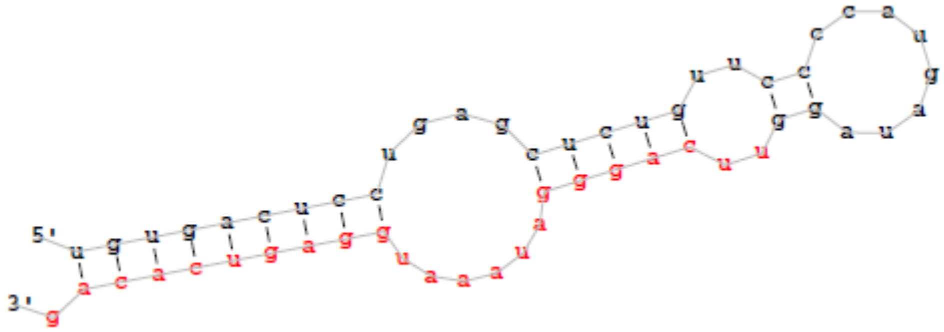
chr9_44662	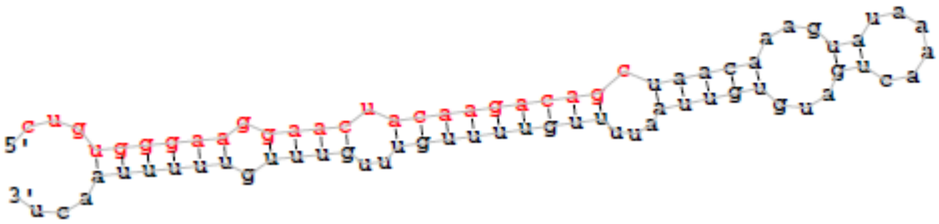
chr11_7315	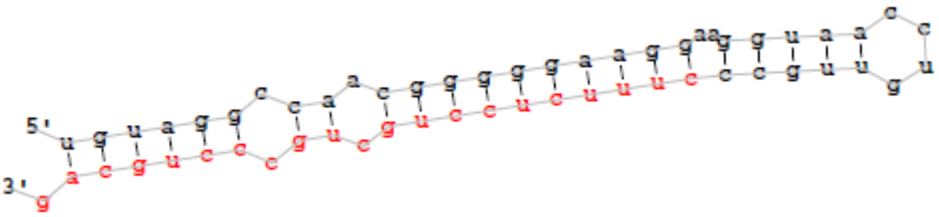

chr12_10577	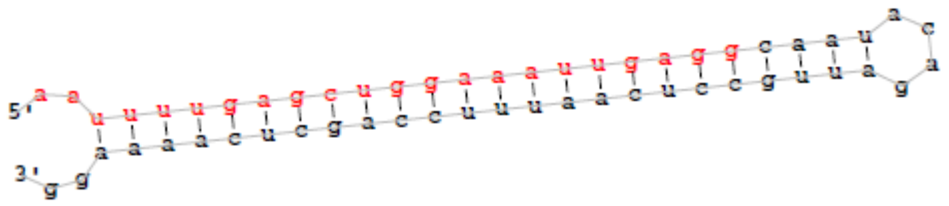
chr14_14675	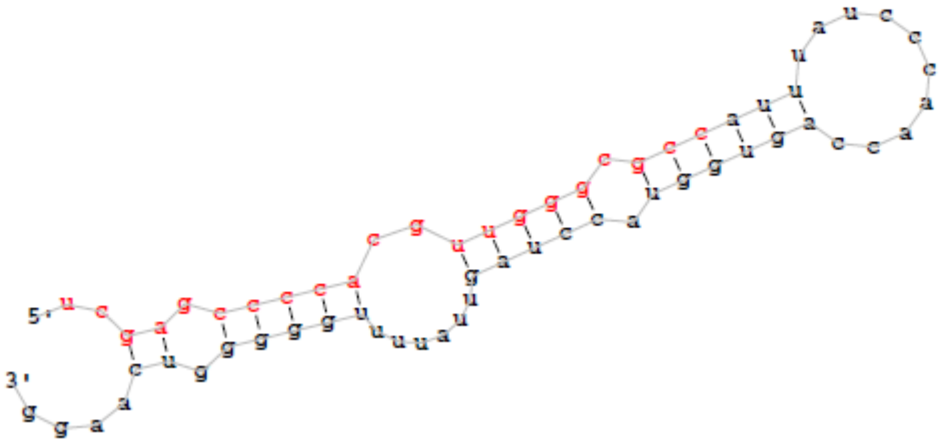
chr14_14933	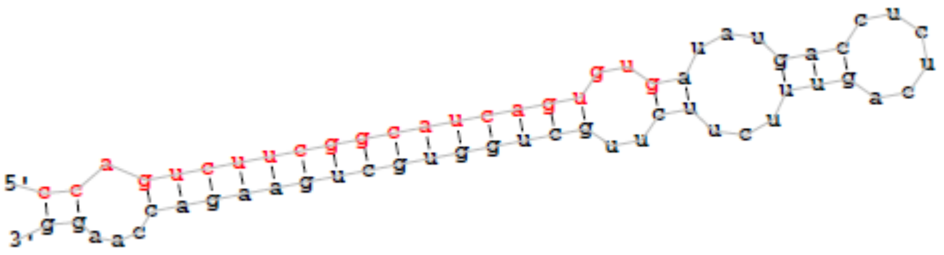
chr14_15012	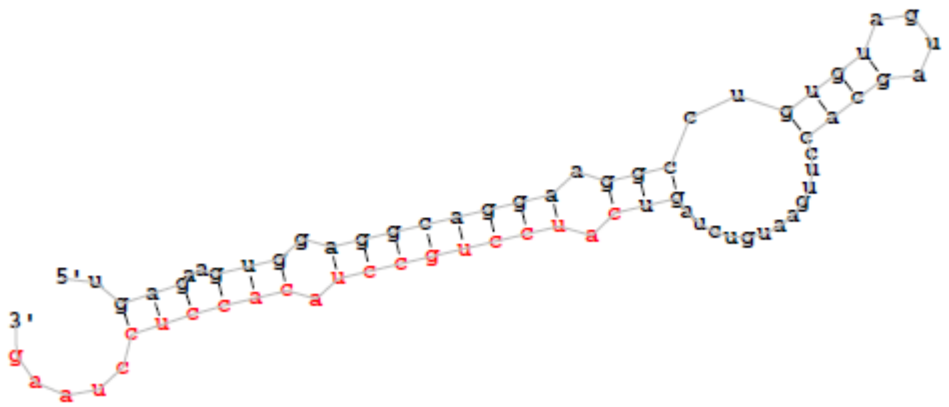

chr15_16817	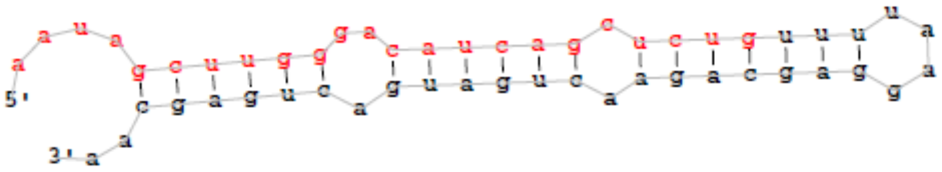
chr17_19864	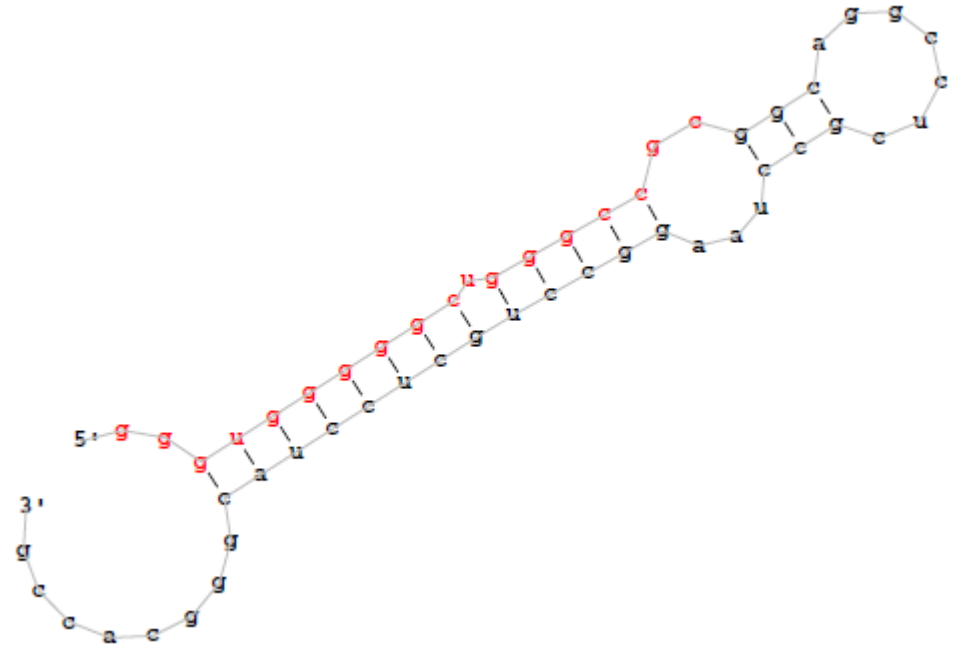
chr18_22209	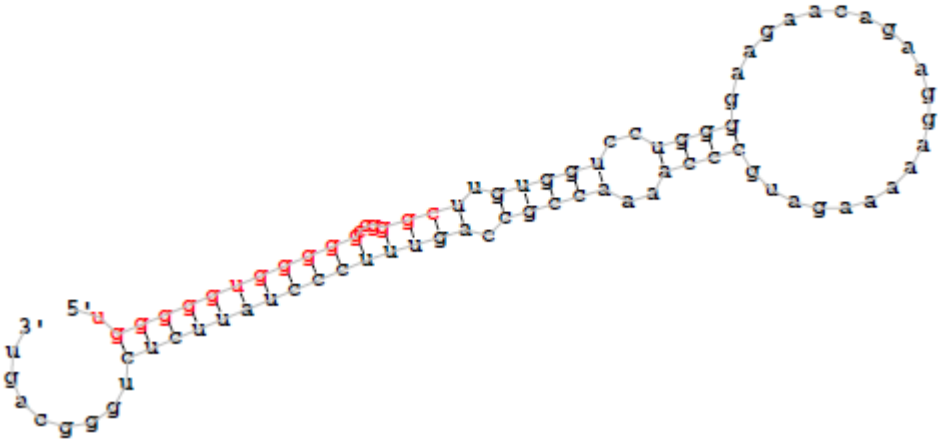

* chr5_33756 has a unique predicted precursor structure for each of its putative genomic coordinates
