# Supplementary material for: Mouse microglia express unique miRNA-mRNA networks to facilitate age-specific functions in the developing central nervous system
Source: Commun Biol. 2023 May 22;6:555. doi: 10.1038/s42003-023-04926-8 (PMC10203306; doi:10.1038/s42003-023-04926-8)
Supplement: Supplementary file 3 — Description of Additional Supplementary Files [file 42003_2023_4926_MOESM3_ESM.pdf]

Description of Additional Supplementary Files

File name: Supplementary Data 1
Description: Microglia vs Bulk differential expression analysis (for each age group).

File name: Supplementary Data 2
Description: miRNA-mRNA network analysis.

File name: Supplementary Data 3
Description: KEGG analysis of miRNA-mRNA network results.

File name: Supplementary Data 4
Description: GO analysis of miRNA-mRNA network results.

File name: Supplementary Data 5
Description: miRNA differential expression analysis (between each age group).

File name: Supplementary Data 6
Description: Male vs Female mRNA differential expression analysis (within each age group).

File name: Supplementary Data 7
Description: Numerical source data.
