# Supplementary material for: Mouse microglia express unique miRNA-mRNA networks to facilitate age-specific functions in the developing central nervous system
Source: Commun Biol. 2023 May 22;6:555. doi: 10.1038/s42003-023-04926-8 (PMC10203306; doi:10.1038/s42003-023-04926-8)
Supplement: Supplementary file 11 — Reporting Summary [file 42003_2023_4926_MOESM11_ESM.pdf]

nature portfolio

Corresponding author(s): Michele D Binder

Last updated by author(s): May 8, 2023

Reporting Summary

Nature Portfolio wishes to improve the reproducibility of the work that we publish. This form provides structure for consistency and transparency in reporting. For further information on Nature Portfolio policies, see our Editorial Policies and the Editorial Policy Checklist.

Statistics

For all statistical analyses, confirm that the following items are present in the figure legend, table legend, main text, or Methods section.

n/a	Confirmed	
☐	☒	The exact sample size (*n*) for each experimental group/condition, given as a discrete number and unit of measurement
☐	☒	A statement on whether measurements were taken from distinct samples or whether the same sample was measured repeatedly
☐	☒	The statistical test(s) used AND whether they are one- or two-sided<br>*Only common tests should be described solely by name; describe more complex techniques in the Methods section.*
☐	☒	A description of all covariates tested
☐	☒	A description of any assumptions or corrections, such as tests of normality and adjustment for multiple comparisons
☐	☒	A full description of the statistical parameters including central tendency (e.g. means) or other basic estimates (e.g. regression coefficient) AND variation (e.g. standard deviation) or associated estimates of uncertainty (e.g. confidence intervals)
☐	☒	For null hypothesis testing, the test statistic (e.g. *F*, *t*, *r*) with confidence intervals, effect sizes, degrees of freedom and *P* value noted<br>*Give P values as exact values whenever suitable.*
☒	☐	For Bayesian analysis, information on the choice of priors and Markov chain Monte Carlo settings
☒	☐	For hierarchical and complex designs, identification of the appropriate level for tests and full reporting of outcomes
☐	☒	Estimates of effect sizes (e.g. Cohen's *d*, Pearson's *r*), indicating how they were calculated

Our web collection on statistics for biologists contains articles on many of the points above.

Software and code

Policy information about availability of computer code

Data collection: N/A

Data analysis:

*** R programming language and modules ***
- R (ver 4.0.0)
- Subread (ver 2.0.0)
- limma (ver 3.48.1)
- edgeR (ver 3.34.0)
- EnhancedVolcano (ver 1.10.0)
- R (ver 4.0.0)
- psych (ver 2.16)
- multimiR (ver 1.140)

*** Other Software ***
- fastp (ver 0.20.0)
- mirDeep2 (ver 0.1.3)
- STAR (ver 2.7.3)
- RNAhybrid (ver 2.2.1)
- Cytoscape (ver 3.9.1)

- Graphpad Prism (ver 9.2.0)

For manuscripts utilizing custom algorithms or software that are central to the research but not yet described in published literature, software must be made available to editors and reviewers. We strongly encourage code deposition in a community repository (e.g. GitHub). See the Nature Portfolio guidelines for submitting code & software for further information.

Data

Policy information about availability of data

All manuscripts must include a data availability statement. This statement should provide the following information, where applicable:

- Accession codes, unique identifiers, or web links for publicly available datasets
- A description of any restrictions on data availability
- For clinical datasets or third party data, please ensure that the statement adheres to our policy

Provide your data availability statement here.

Human research participants

Policy information about studies involving human research participants and Sex and Gender in Research.

Reporting on sex and gender	N/A
Population characteristics	N/A
Recruitment	N/A
Ethics oversight	N/A

Note that full information on the approval of the study protocol must also be provided in the manuscript.

Field-specific reporting

Please select the one below that is the best fit for your research. If you are not sure, read the appropriate sections before making your selection.

[x] Life sciences [ ] Behavioural & social sciences [ ] Ecological, evolutionary & environmental sciences

For a reference copy of the document with all sections, see nature.com/documents/nr-reporting-summary-flat.pdf

Life sciences study design

All studies must disclose on these points even when the disclosure is negative.

Sample size	RNAseq sample size was determined to match or exceed those of similar studies (Kodama et al Nat Neurosci 2020 ; Villa et al Cell Rep 2018), such that comparisons between age groups were 12 vs 12 (matched for sex), and between tissue types were 36 vs 36 (correcting for animal, age and sex). For differential expression analysis we used the empirical Bayes procedure implemented in R limma software to maximise sensitivity to detect differentially expressed genes. To prioritise differences most likely to be biologically relevant, we subsequently applied the limma treat procedure to discard results with an absolute fold change < 2.
Data exclusions	One sample was excluded from sequencing prior to library construction as the RNA failed QC. One biological replicate was excluded from analysis based on Multi Dimensional Scaling Analysis.
Replication	We did not undertake replication, given the complexity of the experiments. Validation of key results from RNA sequencing were validated using qPCR. All validation experiments are included in the manuscript.
Randomization	Randomisation to groups not relevant - all mice were ordered from a commercial animal supplier to match appropriate ages then all were collected
Blinding	Blinding not relevant as we did not have treatment groups.

Reporting for specific materials, systems and methods

We require information from authors about some types of materials, experimental systems and methods used in many studies. Here, indicate whether each material, system or method listed is relevant to your study. If you are not sure if a list item applies to your research, read the appropriate section before selecting a response.

Materials & experimental systems

n/a	Involved in the study	
☐	☒	Antibodies
☒	☐	Eukaryotic cell lines
☒	☐	Palaeontology and archaeology
☐	☒	Animals and other organisms
☒	☐	Clinical data
☒	☐	Dual use research of concern

Methods

n/a	Involved in the study	
☒	☐	ChIP-seq
☒	☐	Flow cytometry
☒	☐	MRI-based neuroimaging

Antibodies

Antibodies used	Anti-CD45 antibody (#550539; BD Pharmingen)
Validation	Routinely tested for mouse reactivity by the manufacturer using flow cytometry.

Animals and other research organisms

Policy information about studies involving animals; ARRIVE guidelines recommended for reporting animal research, and Sex and Gender in Research

Laboratory animals	Mouse C57Bl/6J of multiple ages as outlined in the manuscript
Wild animals	N/A
Reporting on sex	Sex was considered as a variable in this study. The study was appropriately powered to allow for analysis of sex specific differences in miRNA and mRNA transcripts. A section of the results discusses the sex specific analysis outcomes.
Field-collected samples	N/A
Ethics oversight	Animal Ethics Committee at the Florey Institute of Neuroscience and Mental Health.

Note that full information on the approval of the study protocol must also be provided in the manuscript.
